# Computational exploration of anticancer drug adsorption on a porous organic nanocage: Insights from density functional theory for targeted nanocarrier design

**DOI:** 10.1038/s41598-026-51498-8

**Published:** 2026-05-05

**Authors:** Afrasim Moin, Zahrah Ali Asiri, Mona Al Hamod, Noura Al Hamood, Shahad Mari Salem Alshahrani, Farhat Fatima, Ali H. Alamri, Adel Al Fatease, Riyaz Ali M Osmani, Mohamed Rahamathulla, Umme Hani

**Affiliations:** 1 Department of Pharmaceutics, College of Pharmacy, University of Hail, Hail, 81442 Saudi Arabia; 2 Department of Pharmaceutics, College of Pharmacy, King Khalid University, Abha, 62529 Saudi Arabia; 3 Department of Pharmaceutics, Faculty of Pharmacy, Northern Border University, Rafhaa, 73213 Saudi Arabia; 4 Department of Pharmaceutics, College of Pharmacy, Prince Sattam bin Abdulaziz University, Al-Kharj, 11942 Saudi Arabia

**Keywords:** Non-covalent interaction, CC1 nanocage, DFT, Drug delivery, Adsorption, Cancer, Chemistry, Drug discovery, Materials science, Nanoscience and technology

## Abstract

Designing efficient nanocarriers for targeted cancer therapy requires a deep understanding of drug–nanomaterial interactions at the molecular level. In this study, the adsorption behavior of four clinically relevant anticancer drugs, 6-mercaptopurine (6-MP), hydroxyurea (HU), chlormethine (CM), and 5-fluorouracil (5-FU), on a CC1 nanocage was systematically investigated using density functional theory (DFT). Geometry optimizations and thermodynamic analyses were performed at the ωB97XD/6-31G(d, p) level, while optical properties were assessed through time-dependent DFT (TD-DFT) calculations at the B3LYP/6-31G(d, p) level. The computed adsorption energies and electronic descriptors revealed that all drug–nanocage interactions are spontaneous and exothermic in an aqueous medium, with 5-FU exhibiting the strongest binding (− 8.68 kcal/mol). TD-DFT spectra displayed redshifts in absorption peaks after adsorption, signifying charge transfer between the drugs and the CC1 framework. Moreover, topological (QTAIM) and Non-Covalent Interaction (NCI) analyses confirmed that van der Waals forces primarily stabilize the complexes. The short recovery times predicted for the drug-loaded nanocage suggest efficient release kinetics under physiological conditions. Overall, this theoretical study provides a molecular-level perspective on CC1 nanocage–drug interactions, offering useful insights for the rational design of carbon-based nanocarriers in targeted anticancer drug delivery.

**Supplementary Information:**

The online version contains supplementary material available at 10.1038/s41598-026-51498-8.

## Introduction

Targeted drug delivery is a crucial strategy for enhancing therapeutic efficacy while minimizing side effects associated with medications^1–6^. In this context, exploring new carriers for drug loading and release is a vital step toward advancing drug delivery systems, particularly through the development of innovative adsorbents that can optimize drug adsorption processes^7^. Tailoring these carriers and characterizing their interactions with drug molecules can provide valuable insights for selecting the most suitable systems for the targeted delivery of specific drugs. A wide range of materials, such as nanoparticles^8^, liposomes^9^, polymers^10^, and hydrogels^11^, have emerged as versatile drug delivery vehicles, offering precise control over drug release kinetics and enabling more targeted and sustained therapeutic effects.

6-Mercaptopurine (6-MP) is a chemotherapy drug used to treat leukemia^12^. However, its use can lead to various side effects, including loss of appetite, vomiting, liver toxicity, and bone marrow suppression^13^. Hydroxyurea (HU) is employed in the treatment of chronic myelogenous leukemia and various other tumors^14^. Nevertheless, prolonged HU use is associated with several adverse effects, such as scleral hyperpigmentation, alopecia, leg ulcers, and leukopenia. Chlormethine (CM) is used to treat several conditions, including prostate cancer, lymphoid malignancies (e.g., Hodgkin’s disease and lymphosarcoma), chronic myelocytic leukemia, polycythemia vera, and bronchogenic carcinoma^15^. However, at high doses, CM can cause severe side effects, posing risks especially for pregnant or nursing women. 5-Fluorouracil (5-FU) is a well-established anticancer medication commonly prescribed for malignant and benign tumors, including cancers of the breast, colon, skin, head, neck, esophagus, stomach, and pancreas^16^. Despite its efficacy, 5-FU administration can result in unavoidable side effects, such as anemia, diarrhea, and vomiting^17^. Therefore, the development of innovative drug delivery vehicles is essential to mitigate these adverse effects and improve treatment outcomes.

Nanotechnology plays a pivotal role in the advancement of nanocarriers for anticancer medications^18^. These nanocarriers are considered a promising approach for cancer therapy due to their unique physical and chemical properties, which can also enhance the immune response^19–21^. In addition, they improve the solubility and bioavailability of drugs and enable higher therapeutic concentrations at targeted tumor sites while reducing side effects. A wide variety of nanocarriers such as nanotubes^22–26^, nanosheets^27^, nanocages^28–31^, quantum dots^32^, and nanocomposites^33^ have been employed in drug delivery applications. With the ongoing advancement of nanocarrier technology, researchers are increasingly exploring novel materials for targeted drug delivery, largely due to their high surface areas, which make them highly effective adsorbents.

Porous nanostructures have attracted significant attention for their potential applications in adsorption, separation, and catalysis, owing to their high surface areas, tunable pore sizes, and diverse chemical functionalities^34^. A wide range of nanoporous materials exists, including zeolites^35^, covalent organic frameworks (COFs)^36^, metal–organic frameworks (MOFs)^37^, and the more recently developed porous organic cages (POCs)^38^. These porous materials are composed of discrete, covalently bonded organic molecules that possess guest-accessible voids. Among them, POCs have gained considerable attention in recent years due to their high surface-to-volume ratio, substantial porosity, thermal and chemical stability, adjustable pore sizes, and well-defined nanoscale cavities^39^.

Cycloimine cages (CCs) are a fascinating class of molecular constructs that have attracted considerable interest in supramolecular chemistry and materials science^40^. Characterized by their unique cyclic structures, these cages are formed through imine linkages, which yield well-defined three-dimensional architectures. Beyond their structural elegance, CCs possess intrinsic properties such as high stability, porosity, and selectivity, making them promising candidates for hosting guest molecules^41^. Their porous nature allows for the selective uptake of small molecules, ions, and even larger bioactive compounds, a feature essential for applications in nanotechnology and biomedicine.

Several studies have investigated the adsorption of drug molecules onto various fullerenes, nanocages, and nanoclusters using density functional theory (DFT) calculations. However, the literature has primarily focused on non-carbon or heteroatom-doped nanocages as drug carriers. For instance, Gholami et al.^42^ reported that the CM drug interacts with B_24_O_24_ nanocage with favorable adsorption energies of -33.89 kcal/mol in the gas phase and − 31.36 kcal/mol in water, indicating its potential for drug delivery. Saadh et al.^43^ studied the adsorption of the HU drug onto Fe-doped BN nanocages, finding that doping reduced the HOMO-LUMO energy gap and enhanced the drug-nanocage interaction. Yang et al^44^ reported that the B_24_N_24_ nanocage exhibits both physisorption (-2.76 to -4.60 kcal/mol) and chemisorption (-11.28 to -15.65 kcal/mol) mechanisms for drug adsorption. Similarly, Vessally et al.^45^ demonstrated that boron doping significantly enhances the adsorption energy of 5-FU on a C_24_ fullerene to -27.2 kcal/mol.

The use of pristine nanomaterials without structural modifications has emerged as a feasible and cost-effective strategy for delivering anticancer drugs, as it helps reduce degradation and minimize unwanted interactions with biological molecules^46,47^. Their inherent surface chemistry, combined with high surface area and stability, makes pristine nanomaterials a promising platform for the development of next-generation drug delivery systems. The CC1 nanocage is a three-dimensional, shape-persistent chiral cage capable of forming crystalline materials with tunable porosity^40^. The reactivity within such porous organic cages provides an excellent platform for adsorbing guest molecules. The CC1 cage, featuring nitrogen atoms, offers an electron-rich porous environment that enhances drug adsorption efficiency.

To the best of our knowledge, the potential of the CC1 nanocage as a platform for drug delivery has not yet been explored. Motivated by this, we investigated the adsorption behavior of the anticancer drugs 6-MP, HU, CM, and 5-FU on the CC1 nanocage using DFT calculations. This analysis included geometry optimization, adsorption energy and thermodynamic evaluations, UV–Vis spectra, density of states (DOS), electron density differences (EDD), and charge decomposition analysis (CDA). Furthermore, quantum theory of atoms in molecules (QTAIM) and non-covalent interaction (NCI) analyses were conducted to characterize the nature and strength of the intermolecular interactions between the drugs and the nanocage. The results indicate that all studied drugs can be accommodated within the CC1 cavity, demonstrating that the nanocage possesses sufficient porosity and structural stability for effective adsorption. These findings highlight the potential of CC1 as a nanoscale carrier capable of improving the delivery efficiency and selectivity of anticancer agents. Furthermore, the insights gained from these analyses provide a comprehensive understanding of the thermodynamic, electronic, and structural factors governing drug–nanocage interactions, offering guidance for the rational design of CC1-based drug delivery systems.

### Computational methods

All theoretical calculations were performed using density functional theory (DFT) with the Gaussian 16 package^48^; optimized structures were visualized using GaussView 6.0.16^49^. All isolated species (CC1 nanocage and drug molecules) as well as the CC1@drug complexes were considered in their neutral charge state (total charge = 0) and singlet spin multiplicity (M = 1), corresponding to their ground electronic states. Geometry optimizations were conducted in an aqueous environment (water) using the ωB97XD functional and the 6-31G(d, p) basis set. Water was chosen to mimic physiological conditions, as it is the primary biological solvent in which drug–nanocage interactions occur. The ωB97XD functional is a range-separated hybrid functional that incorporates empirical dispersion corrections and has been widely validated for accurately describing non-covalent interactions, long-range charge transfer effects, and thermochemical properties in supramolecular and adsorption systems.^50–52^. The 6-31G(d, p) basis set was also selected as a balance between computational cost and accuracy, and has been successfully employed in many previous DFT studies of drug–nanocarrier systems^53–57^.

To ensure that the optimized structures correspond to true minima, vibrational frequency calculations were performed at the same level of theory to confirm the absence of imaginary frequencies. The adsorption energy (E_ads_) of the selected drugs (6-MP, HU, CM, and 5-FU) onto the CC1 nanocage was computed using the following equation:

1 $${\mathrm{E}}_{{{\mathrm{ads}}}} = {\text{ E}}_{{{\mathrm{CC1}}@{\mathrm{drug}}}} ~\left( {{\mathrm{E}}_{{{\mathrm{CC1}}}} + {\text{ E}}_{{{\mathrm{drug}}}} } \right)$$

Here, the adsorption energies of the complex, nanocage, and drug are denoted as E_CC1@drug_, E_CC1_, and E_drug_, respectively. To account for basis set superposition error (BSSE), the counterpoise correction method⁵⁸ was employed to calculate more accurate adsorption energies, as given by the following equation:

2 $${\mathrm{E}}_{{{\mathrm{ads}}({\mathrm{corrected}})}} = {\text{ E}}_{{{\mathrm{ads}}}} + {\text{ E}}_{{{\mathrm{BSSE}}}}$$

where E_ads(corrected)_ is the BSSE-corrected adsorption energy and E_BSSE_ is the basis set superposition error.

To quantify the structural deformation of the CC1 nanocage induced by drug adsorption, the deformation energy (E_def_) for each CC1@drug complex was calculated using the following Eq. 5^9^:

The thermodynamic parameters, including the changes in Gibbs free energy (ΔG), enthalpy (ΔH), and entropy (ΔS), were calculated to evaluate the thermodynamic stability and spontaneity of the adsorption process using the following equations:

3 $${\mathrm{E}}_{{{\mathrm{def}}}} = {\text{ E}}_{{{\mathrm{CC1}}}} {-}{\text{ E}}_{{{\mathrm{CC1}}}} {\text{in complex}}$$

4 $$\Delta {\text{G }} = {\text{ G}}_{{{\mathrm{CC1}}@{\mathrm{drug}}}} ~\left( {{\mathrm{G}}_{{{\mathrm{CC1}}}} + {\text{ G}}_{{{\mathrm{drug}}}} } \right)$$

5 $$\Delta {\text{H }} = {\text{ H}}_{{{\mathrm{CC1}}@{\mathrm{drug}}}} ~\left( {{\mathrm{H}}_{{{\mathrm{CC1}}}} + {\text{ H}}_{{{\mathrm{drug}}}} } \right)$$

6 $$\Delta{S}=\:\frac{{\Delta\:}\mathrm{H}-{\Delta\:}\mathrm{G}}{\mathrm{T}}$$.

The electronic properties of the CC1@drug complexes were investigated using frontier molecular orbital (FMO) theory. The energies of the highest occupied and lowest unoccupied molecular orbitals (E_HOMO_ and E_LUMO_, respectively) and the HOMO–LUMO energy gap (E_g_) were determined at the same level of theory. Global reactivity descriptors, including chemical hardness (η), chemical potential (µ), and electrophilicity (ω), were calculated according to Koopmans’ theorem^60^ using the following equations:

7 $$\:{\upeta\:}\:=\:\frac{{\mathrm{E}}_{\mathrm{H}\mathrm{O}\mathrm{M}\mathrm{O}}+\:{\mathrm{E}}_{\mathrm{L}\mathrm{U}\mathrm{M}\mathrm{O}}\:}{2}$$

8 $$\mu {\text{ }} = \:\frac{{\mathrm{E}}_{\mathrm{L}\mathrm{U}\mathrm{M}\mathrm{O}}-\:{\mathrm{E}}_{\mathrm{H}\mathrm{O}\mathrm{M}\mathrm{O}}\:}{2}$$

9 $$\omega=\:\frac{{{\upmu\:}}^{2}}{2{\upeta\:}}$$

It is important to note that Koopmans’ theorem provides an approximation for ionization potential and electron affinity from HOMO and LUMO energies, but it neglects electron correlation and orbital relaxation effects. Although experimental data are unavailable for these specific CC1@drug complexes, this approach is well-established for analyzing comparative trends in electronic reactivity and for evaluating differences among the complexes studied.

The percentage change in the HOMO-LUMO energy gap (%ΔE_g_) due to the interaction between the CC1 nanocage and the drug molecules was calculated using the following equation:

10 $$\:{\%}{\Delta\:}\mathrm{E}\mathrm{g}\:=\:\frac{{\mathrm{E}}_{\mathrm{g}}(\mathrm{C}\mathrm{C}1@\mathrm{d}\mathrm{r}\mathrm{u}\mathrm{g})\:-\:{\mathrm{E}}_{\mathrm{g}}\left(\mathrm{C}\mathrm{C}1\right)}{{\mathrm{E}}_{\mathrm{g}}(\mathrm{C}\mathrm{C}1@\mathrm{d}\mathrm{r}\mathrm{u}\mathrm{g})}\:\times\:100$$

The recovery time (τ) is the theoretical duration required for an adsorbed drug molecule to desorb from the nanocarrier surface^61^. This parameter is a key metric for evaluating the efficiency of drug release. The release kinetics can be predicted from the adsorption energy (E_ads_), where a less negative E_ads_ indicates a weaker drug-carrier interaction and consequently facilitates faster desorption. The recovery time was calculated using the following Eqs. 6^2–64^:

11 $$τ = υ_0^(-1)exp(〖-E〗_ads/(K_B T))$$

In this equation, υ_0_ and K_B_ represent the attempt frequency (10¹² s^− 1^) and the Boltzmann constant (1.98 × 10^− 3^ kcal/mol K), respectively.

To gain quantitative insight into the charge-transfer interactions between the drugs and the CC1 nanocage, Natural Bond Orbital (NBO) analysis was performed at the same level of theory used for the geometry optimization. The polarizable continuum model (PCM)^65^ was employed to simulate the aqueous solvation effects on the adsorption of drugs onto the CC1 nanocage. The nature of the non-covalent interactions was investigated using Quantum Theory of Atoms in Molecules (QTAIM) and Non-Covalent Interaction (NCI) analyses. These analyses were performed using the Multiwfn 3.8^66^ and VMD 1.9.3^67^ software packages. Time-dependent density functional theory (TD-DFT) calculations at the B3LYP/6-31G(d, p) level of theory were conducted to simulate the UV–Vis spectra; the spectra were generated using the GaussSum 2.2.0 program^68^. The B3LYP functional was selected based on its established accuracy for predicting absorption spectra and providing reliable excitation energies, as demonstrated in previous studies^69–71^.

## Results and discussion

### Optimized structures and stability parameters

**Fig. 1:**
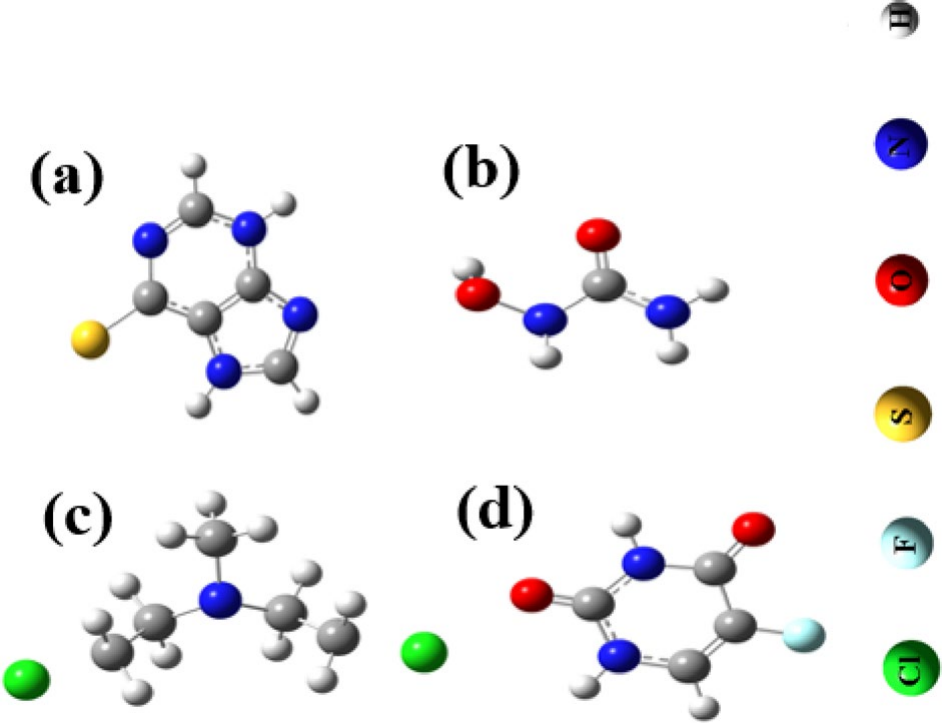
Optimized molecular structures of the studied drugs: (**a**) 6-mercaptopurine (6-MP), (**b**) hydroxyurea (HU), (**c**) chlormethine (CM), and (**d**) 5-fluorouracil (5-FU).

**Fig. 2:**
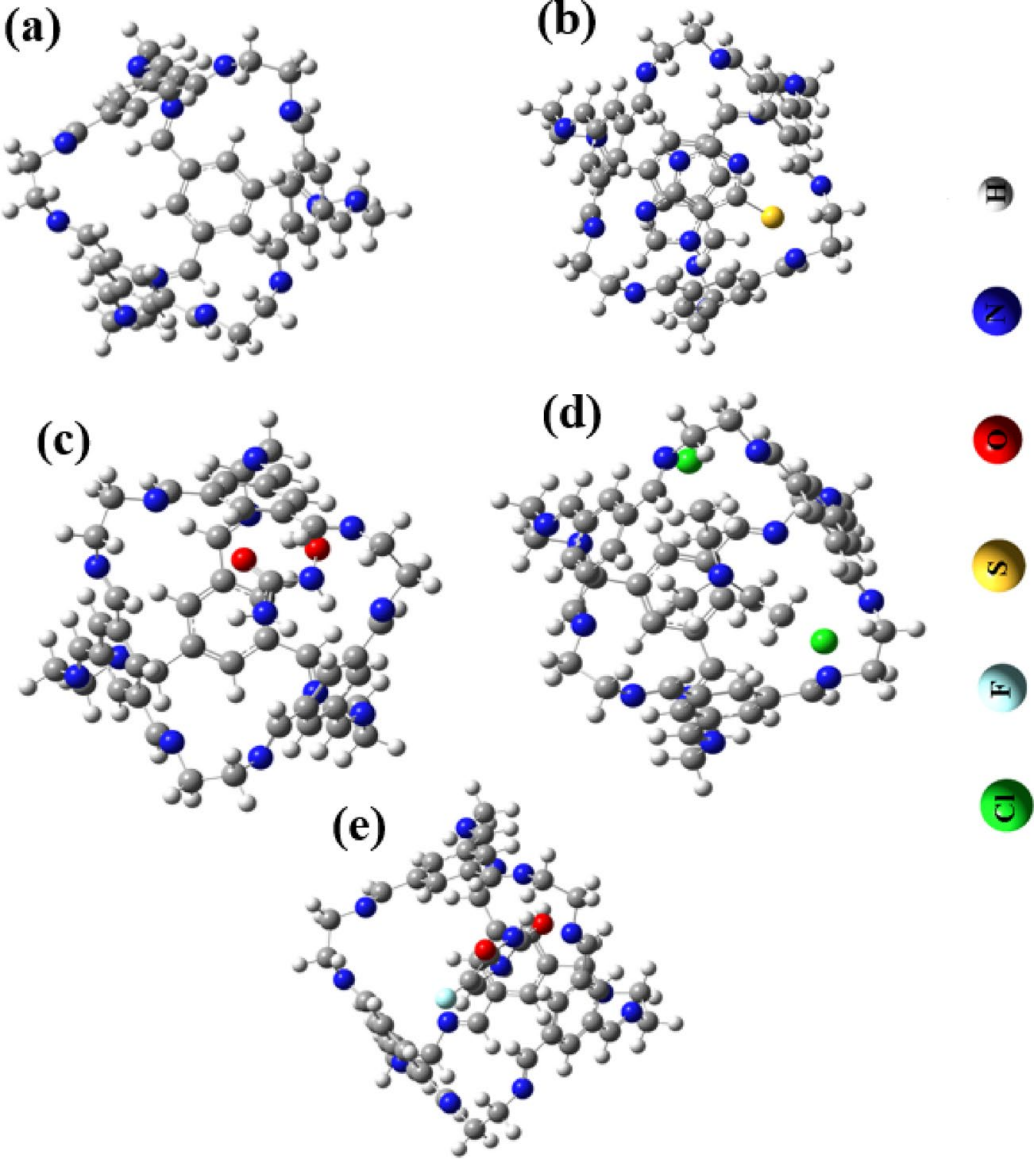
Optimized geometries of (**a**) CC1, (**b**) CC1@6-mercaptopurine (6-MP), (**c**) CC1@hydroxyurea (HU), (**d**) CC1@chlormethine (CM), and (**e**) CC1@5-fluorouracil (5-FU).

The optimized geometries of the studied drug molecules and the corresponding CC1@drug complexes are presented in Figs. 1 and 2, respectively. For chlormethine (CM), the optimized C–Cl bond length is calculated to be 1.90 Å, which is slightly longer than a typical C–Cl bond (~ 1.77 Å), indicating slight bond elongation. This elongation can be attributed to steric and electronic effects arising from adsorption inside the cavity of the CC1 nanocage; however, the bond remains intact without any dissociation upon encapsulation. For each system, a single initial configuration was considered in which the drug molecule was positioned inside the cavity of the CC1 nanocage, corresponding to endohedral adsorption (encapsulation). This mode of adsorption is particularly significant for drug delivery applications, as it ensures enhanced protection of the drug from premature degradation, improves stability within the biological environment, and enables controlled and sustained release. Encapsulation also promotes stronger host–guest interactions and effective confinement of the drug molecule, which can increase loading efficiency and optimize release kinetics, making it a critical feature for designing efficient nanocarrier systems.

All geometries were fully optimized without symmetry constraints, and subsequent vibrational frequency analyses confirmed that the optimized structures correspond to true minima on the potential energy surface, as no imaginary frequencies were observed. Structural analysis indicates that adsorption inside the cavity does not induce significant deformation of the CC1 nanocage, demonstrating its structural robustness. As summarized in Table 1, the calculated deformation energies for all complexes are small (ranging from − 0.55 to − 2.07 kcal/mol), indicating only minor conformational adjustments during the encapsulation (endohedral adsorption) process.

**Table 1:** Deformation energy (E_def_), adsorption energy (E_ads_), basis set superposition error (BSSE), corrected adsorption energy (E_ads(corrected)_), change in Gibbs free energy (ΔG), change in enthalpy (ΔH), and change in entropy (ΔS) for CC1@drug complexes.

Complex	E_def_ (kcal/mol)	E_ads_ (kcal/mol)	BSSE (kcal/mol)	E_ads(corrected)_ (kcal/mol)	ΔH (kcal/mol)	ΔG (kcal/mol)	ΔS (kcal/mol K)
CC1@6-MP	-0.94	-5.64	0.014	-5.62	-6.96	-4.39	-0.008
CC1@HU	-1.98	-7.46	0.012	-7.44	-8.03	-6.21	-0.006
CC1@CM	-0.55	-2.51	0.010	-2.50	-3.73	-3.01	-0.002
CC1@5-FU	-2.07	-8.70	0.014	-8.68	-10.76	-9.36	-0.004

### HOMO-LUMO energy gap

The stability and reactivity of the CC1 nanocage for drug delivery applications were evaluated using frontier molecular orbital (FMO) theory. A key parameter in this analysis is the HOMO–LUMO energy gap (E_g_). Variations in E_g_ can induce significant exponential changes in electrical conductivity^73^. A smaller E_g_ is generally associated with higher chemical reactivity, greater sensitivity, and improved electrical conductivity. Table 2 summarizes the key electronic parameters, E_HOMO_, E_LUMO_, E_g_, and its percentage change. The E_g_ calculated for the isolated CC1 nanocage is 3.10 eV. This value is lower than a previously reported value of 5.03 eV^40^, a discrepancy we attribute to the inclusion of solvent effects in our model. The presence of a polar aqueous environment stabilizes the frontier orbitals, reducing the band gap and thereby enhancing the nanocage chemical reactivity. This effect is highly relevant for drug delivery applications, where interactions occur in aqueous biological media.

Following drug adsorption, the E_g_ decreases to 2.78, 2.04, 1.70, and 1.59 eV for the CC1@6-MP, CC1@HU, CC1@CM, and CC1@5-FU complexes, respectively. This reduction in E_g_ indicates an increase in the chemical reactivity and electrical conductivity of the nanocage–drug complexes. The CC1@5-FU complex exhibits the most substantial decrease in E_g_ (48.70%), which correlates strongly with its having the most negative adsorption energy (E_ads_ = -8.70 kcal/mol, Table 1). This strong correlation indicates that the most stable adsorption (strongest binding) is associated with the most significant enhancement of electronic properties. This suggests that 5-FU forms the most stable and effective complex with the CC1 nanocage, supporting the potential of CC1 as a nanocarrier for this drug.

**Fig. 3:**
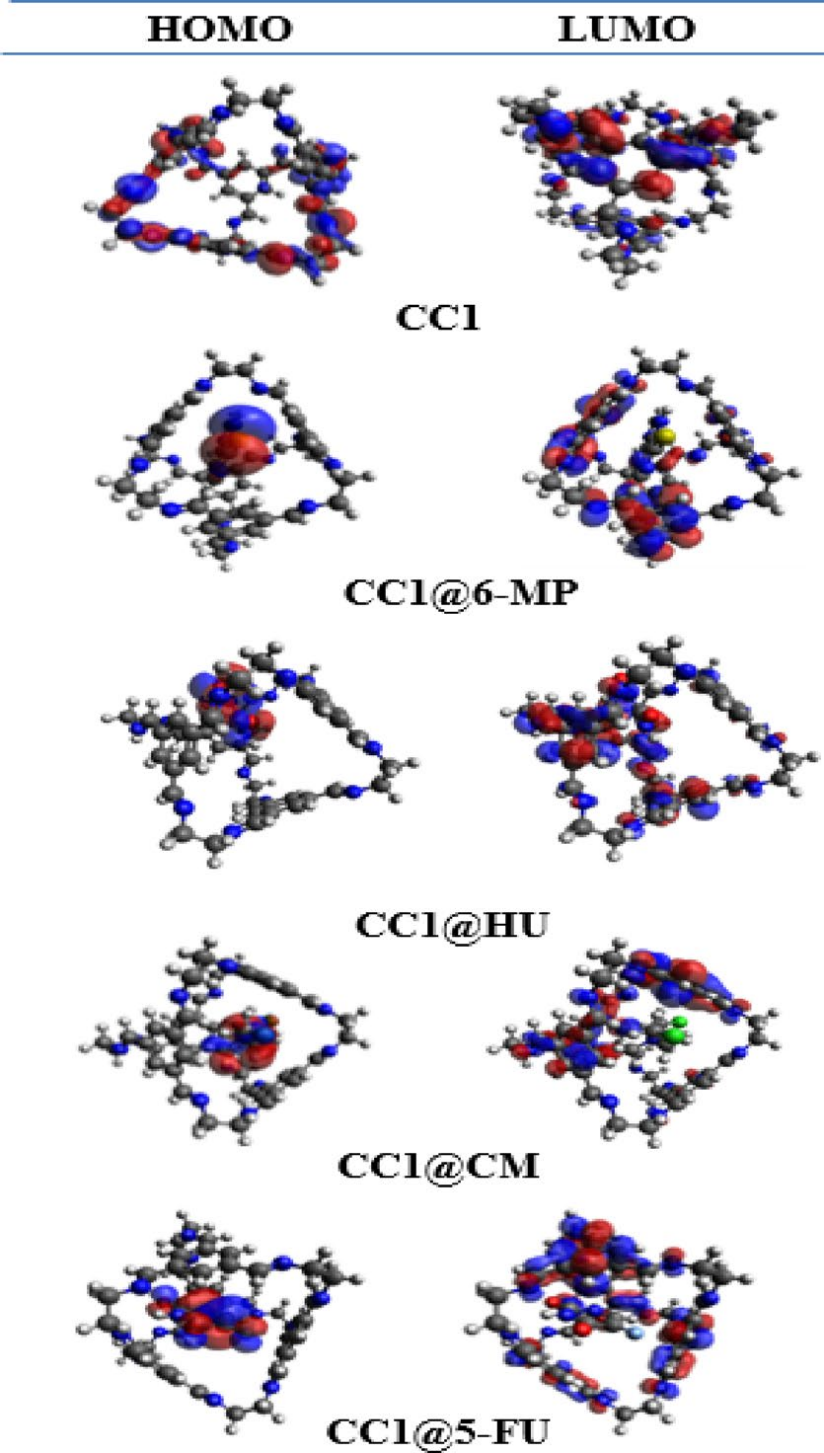
Frontier molecular orbital distributions for the CC1 nanocage and CC1@drug complexes.

The distributions of the frontier molecular orbitals are illustrated in Fig. 3. In the isolated CC1 nanocage, the HOMO is primarily localized on the molecular periphery, while the LUMO is concentrated near its center. Following adsorption, a notable shift in the orbital distribution is observed: the LUMO remains predominantly on the nanocage, whereas the HOMO is primarily located on the adsorbed drug molecule. This redistribution suggests significant electron delocalization and charge transfer from the nanocage to the drug within the complexes.

**Table 2:** Energy of HOMO (E_HOMO_), energy of LUMO (E_LUMO_), HOMO-LUMO energy gap (E_g_), and percentage change in HOMO-LUMO energy gap (%ΔE_g_) for CC1 nanocage and CC1@drug complexes.

Compound	E_HOMO_ (eV)	E_LUMO_(eV)	E_g_(eV)	%ΔE_g_
CC1	-7.11	-4.01	3.10	-
CC1@6-MP	-9.38	-6.60	2.78	-10.32
CC1@HU	-8.09	-6.05	2.04	-34.19
CC1@CM	-8.15	-6.45	1.70	-45.16
CC1@5-FU	-8.31	-6.72	1.59	-48.70

### DOS analysis

To evaluate the influence of drug adsorption on the electronic properties of the nanocage, a detailed density of states (DOS) analysis was performed. The projected density of states (PDOS) was calculated for both pristine CC1 and CC1@drug complexes using a fragment-based approach, with explicit resolution of S and P orbital contributions. For pristine CC1, the PDOS reflects the intrinsic electronic structure of the nanocage, showing distinct S and P orbital distributions. Upon drug adsorption, the PDOS of the complexes reveals clear orbital hybridization between the CC1 nanocage and the adsorbed molecules, manifested as an overlap between the S orbitals of CC1 and the P orbitals of the drug species, indicating electronic interaction and the formation of new hybrid states. The extent of this S–P orbital overlap varies among the systems and is consistent with adsorption strength. In particular, the CC1@5-FU complex exhibits the most pronounced orbital overlap, in agreement with its highest adsorption energy (-8.70 kcal/mol), confirming the strongest interaction among all studied drugs, while the other complexes show comparatively weaker S–P hybridization corresponding to lower adsorption energies.

**Fig. 4:**
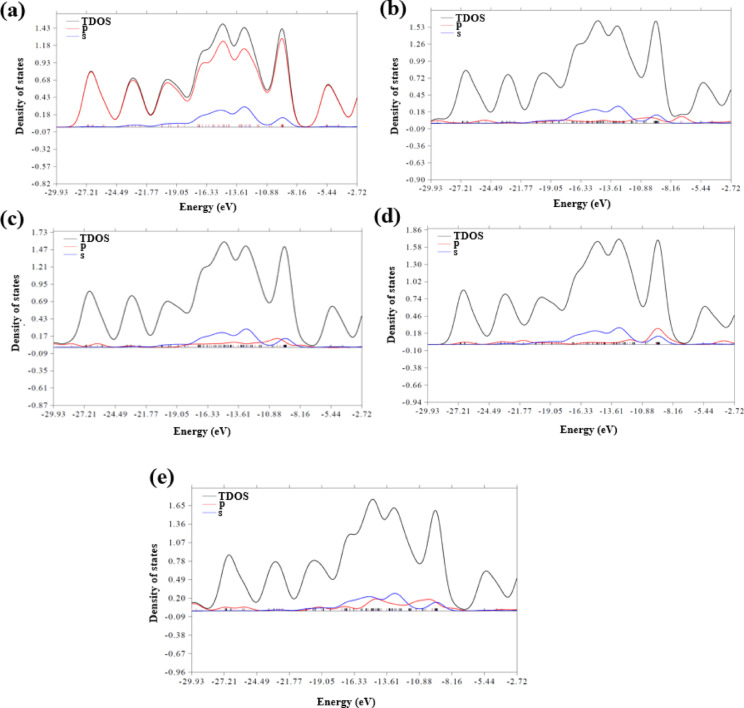
Graphical representation of total and projected density of states (TDOS and PDOS) plots for CC1@drug complexes: (**a**) CC1@6-MP, (**b**) CC1@HU, (**c**) CC1@CM, and (**d**) CC1@5-FU.

### Global reactivity descriptors

Global reactivity descriptors, chemical potential (µ), chemical hardness (η), and electrophilicity (ω), were calculated to evaluate the effect of drug adsorption on the reactivity and selectivity of the CC1 nanocage. As shown in Table 3, the chemical hardness (η) significantly decreases following drug adsorption, indicating that the resulting complexes are chemically softer and more reactive. Furthermore, all CC1@drug complexes exhibit negative chemical potential (µ) values, confirming increased thermodynamic stability and a spontaneous adsorption process. The concomitant rise in electrophilicity (ω) indicates that the complexes are more electrophilic than the pristine nanocage and thus more prone to accept electrons. Collectively, these reactivity descriptors demonstrate that the CC1 nanocage has high sensitivity and a strong affinity for the drug molecules, facilitating efficient interactions.

**Table 3:** Chemical hardness (η), chemical potential (μ), and electrophilicity (ω) for CC1 nanocage and CC1@drug complexes.

Compound	η (eV)	μ(eV)	ω (eV)
CC1	1.55	-5.56	9.97
CC1@6-MP	1.39	-7.99	22.96
CC1@HU	1.02	-7.07	24.50
CC1@CM	0.85	-7.30	31.34
CC1@5-FU	0.79	-7.51	35.69

### EDD analysis

**Fig. 5:**
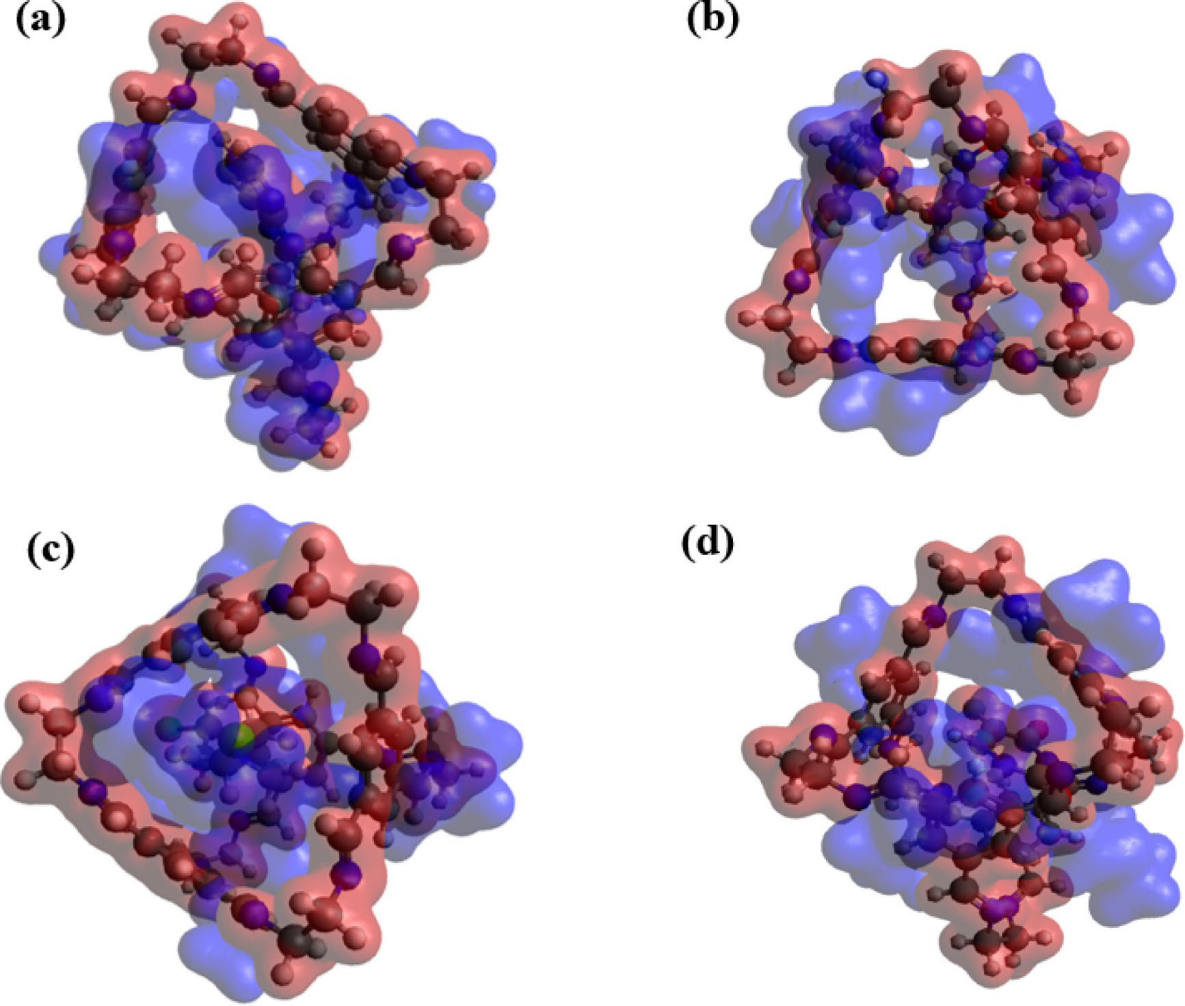
Electron density difference (EDD) isosurfaces for CC1@drug complexes: (**a**) CC1@ 6-MP, (**b**) CC1@HU, (**c**) CC1@CM, and (**d**) CC1@5-FU.

Electron density difference (EDD) analysis was employed to visualize the redistribution of electron density upon complex formation between the drug molecules and the CC1 nanocage. The EDD isosurfaces (Fig. 5), generated by subtracting the sum of the electron densities of the isolated species from the density of the CC1@drug complex, reveal regions of electron accumulation (red) and depletion (blue). The plots show a substantial electron transfer from the drug molecules to the nanocage. The nanocage framework is predominantly surrounded by electron accumulation (red), while the drug molecules are associated with regions of electron depletion (blue). This redistribution confirms that the nanocage acts as an electron acceptor and the adsorbed drugs as electron donors. These results are in excellent agreement with the charge transfer analysis, which consistently showed negative values for all complexes.

### NBO analysis and charge-transfer interactions

To provide quantitative insight into the electronic interactions observed in the EDD analysis, Natural Bond Orbital (NBO) calculations were performed for all CC1@drug complexes, and the dominant donor–acceptor interactions together with their corresponding second-order perturbation stabilization energies (E²) are summarized in Table 4. The NBO results indicate that the primary stabilization mechanism arises from lone pair (LP) → antibonding orbital (BD*) interactions between heteroatoms of the nanocage and hydrogen atoms of the drugs. For CC1@HU, the strongest donor–acceptor interaction is characterized by an E² value of 18.34 kcal/mol corresponding to LP(N1) → BD*(O–H) interaction, reflecting significant charge transfer and pronounced hydrogen-bond-assisted stabilization, which explains the favorable adsorption energy of this complex. In the CC1@6-MP complex, a notable LP(N1) → BD*(N–H) interaction with an E² value of 10.27 kcal/mol is observed, indicating moderate yet meaningful electronic delocalization between the drug and the nanocage. For CC1@CM, the stabilization energy is considerably smaller (E² = 1.70 kcal/mol), suggesting weaker donor–acceptor interaction, in agreement with its relatively lower adsorption energy and minor spectral shifts. In contrast, CC1@5-FU exhibits a very small stabilization energy (E² = 0.08 kcal/mol), indicating minimal orbital overlap and limited charge-transfer contribution despite its favorable adsorption energy, implying that dispersion and electrostatic interactions predominantly govern the stabilization in this system. Overall, the NBO analysis quantitatively corroborates the EDD findings by confirming the presence of charge-transfer interactions between the drugs and the CC1 nanocage; the magnitude of the E² values correlates with interaction strength and provides deeper insight into the electronic stabilization mechanisms responsible for complex formation, reinforcing the non-covalent nature of adsorption and supporting the suitability of CC1 as a potential drug delivery carrier.

**Table 4:** Donor–acceptor interactions and corresponding second-order perturbation energies (E²) obtained from NBO analysis for the CC1@drug complexes.

Compound	Donor	Acceptor	E^2^ (kcal/mol)
CC1@6-MP	LP(N_1_)	BD^*^(N_115_ H_118_)	10.27
CC1@HU	LP(N_1_)	BD^*^(O_113_ H_114_)	18.34
CC1@CM	LP(N_1_)	BD^*^(C_114_ H_124_)	1.70
CC1@5-FU	LP(N_1_)	BD^*^(N_116_ H_117_)	0.08

### IR spectra analysids

Infrared (IR) spectroscopy is a powerful tool for probing intermolecular interactions and confirming complex formation, as vibrational frequency shifts directly reflect changes in bond strength and the local electronic environment. Comparing the IR spectra before and after drug adsorption provides clear evidence of nanocage–drug interactions. The calculated IR spectra of the pristine CC1 nanocage and the CC1@drug complexes are shown in Fig. S1 Both the IR and electronic (UV–Vis) spectra exhibit consistent trends upon complex formation. In the IR spectrum, the C = N stretching mode of CC1 at 1887 cm⁻¹ shifts to lower wavenumbers, indicating weakening of the imine bond due to electronic delocalization and intermolecular interactions. Additionally, in the 6-MP complex, a sharp band appears in the 3500–3600 cm⁻¹ region, corresponding to the N–H stretching vibration, which supports the involvement of non-covalent interactions. Collectively, these systematic frequency shifts and intensity changes confirm successful drug encapsulation and demonstrate that adsorption occurs predominantly via non-covalent interactions, highlighting the potential of the CC1 nanocage as a reversible drug delivery carrier.

### UV-Vis analysis

**Fig. 6:**
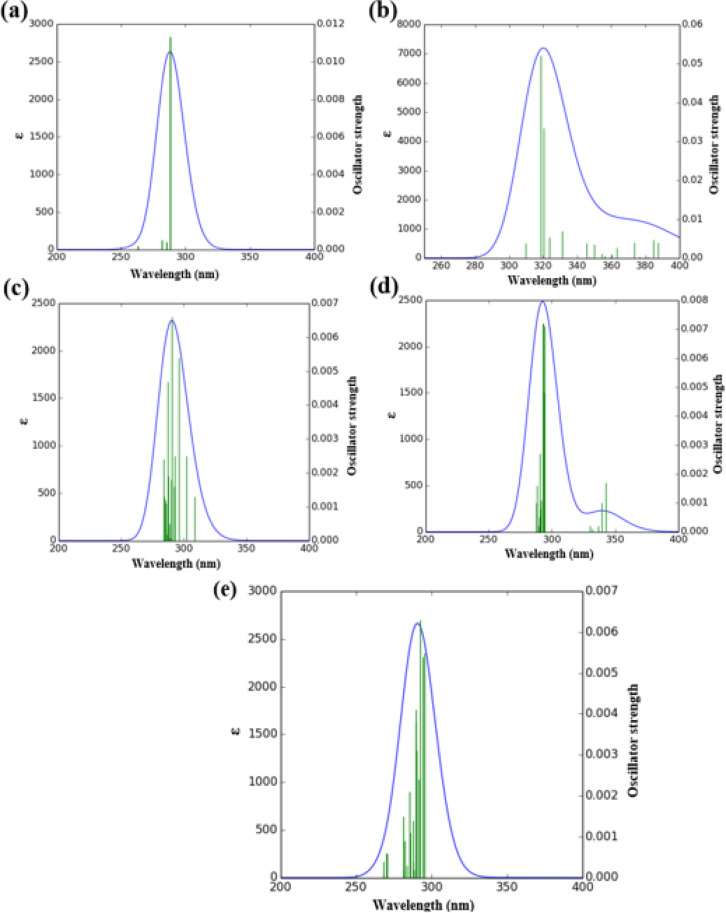
Graphical representation of UV-Vis spectra for (**a**) CC1 (**b**) CC1@6-MP, (**c**) CC1@HU, (**d**) CC1@CM, and (**e**) CC1@5-FU.

To gain deeper insight into the electronic interactions, the UV-Vis absorption spectra of the pristine CC1 nanocage and its drug-loaded complexes were analyzed (Fig. 6). Key optical parameters, the maximum absorption wavelength (λ_max_), excitation energy (E_exc_), and oscillator strength (f), are summarized in Table 5. The pristine CC1 nanocage exhibits an absorption maximum at 288.39 nm (E_exc_ = 4.29 eV). Upon drug complexation, a noticeable red shift is observed in the spectra, evidenced by an increase in λ_max_ and a concomitant decrease in E_exc_. This bathochromic shift indicates a reduction in the HOMO–LUMO energy gap following drug adsorption, signifying enhanced charge-transfer character and stronger electronic coupling. Among the complexes, CC1@6-MP shows the most pronounced shift, with λ_max_ red-shifting to 318.56 nm and E_exc_ decreasing to 3.89 eV. This substantial change suggests particularly strong electronic interactions and efficient intramolecular charge transfer (ICT) between the nanocage and 6-MP.

**Table 5:** Maximum absorption wavelength (λ_max_), excitation energy (E_exc_), oscillation strength (f), and main transitions for CC1 nanocage and CC1@drug complexes.

Complex	λ_max_ (nm)	E_exc_ (eV)	f	Main transitions
CC1	288.39	4.29	0.011	H-15$\:\to\:$LUMO (3%) H-15→L + 4 (3%) H-14→L + 2 (2%) H-14→L + 3 (3%)
CC1@6-MP	318.56	3.89	0.051	H-2→LUMO (23%) H-1→LUMO (19%) H-1→L + 4 (11%)
CC1@HU	290.41	4.26	0.006	H-8→L + 3 (12%) H-8→L + 4 (18%) H-15→L + 10 (2%) H-12→L + 2 (8%)
CC1@CM	293.37	4.22	0.007	H-3→L + 4 (10%) H-13→L + 4 (2%) H-11→L + 1 (2%)
CC1@5-FU	294.22	4.21	0.005	H-10→L + 1 (12%) H-7→L + 1 (23%) H-19→L + 1 (6%) H-17→LUMO (3%)

### QTAIM analysis

**Fig. 7:**
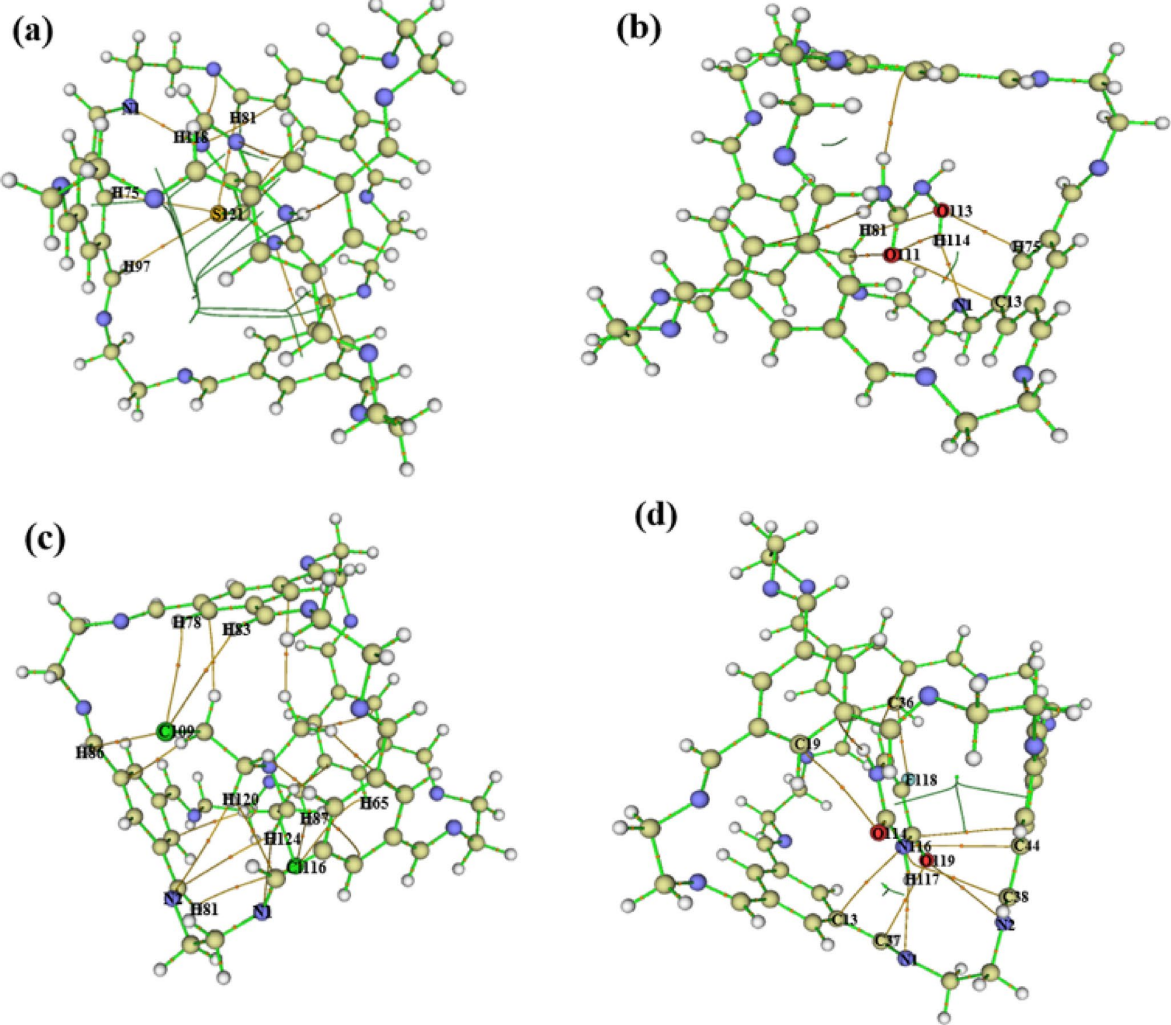
Quantum theory of atoms in molecules (QTAIM) molecular graph of (**a**) CC1@6-MP, (**b**) CC1@HU, (**c**) CC1@CM, and (**d**) CC1@5-FU.

The quantum theory of atoms in molecules (QTAIM) was employed to characterize the nature of the interactions between the CC1 nanocage and the drug molecules. Key topological parameters were evaluated at the bond critical points (BCPs), including the electron density (ρ(r)), the Laplacian of electron density (∇²ρ(r)), kinetic energy density (G(r)), potential energy density (V(r)), and the total energy density (H(r) = G(r) + V(r))^74^. The locations of the BCPs are illustrated in the topological graphs in Fig. 7, and the corresponding numerical values are summarized in Table 6. The results reveal that for all studied complexes, the ∇²ρ(r) and the H(r) at the BCPs are positive. This indicates that the interactions between the nanocage and the drugs are dominated by closed-shell, non-covalent interactions, such as van der Waals forces or electrostatic attractions. This conclusion is further supported by the ratio |G(r)|/|V(r)| (-G(r)/V(r)), a key indicator of bonding nature^75^. Specifically, |G(r)|/|V(r)| > 1 is characteristic of non-covalent interactions, values between 0.5 and 1 suggest intermediate character (e.g., strong electrostatic or weak covalent), and ratios < 0.5 are associated with covalent bonding. For all complexes in this study, the |G(r)|/|V(r)| values were consistently greater than 1, confirming the non-covalent nature of the interactions. These QTAIM findings are consistent with the conclusions drawn from the electron density and Laplacian data. They reinforce that drug adsorption is governed by weak, reversible intermolecular forces, a crucial characteristic for efficient drug loading and release in delivery applications.

**Table 6:** Topological parameters (in a.u) for intermolecular interactions in CC1@drug complexes.

Complex	Bond	ρ(*r*)	$\:{\nabla\:}^{2}\boldsymbol{\uprho\:}$(*r*)	H(*r*)	G(*r*)	V(*r*)	-G(*r*)/V(*r*)
CC1@6-MP	S121$\:-$H75 S121$\:-$H97 S121$\:-$H81 N1$\:-$H118	0.042 0.034 0.044 0.329	0.124 0.099 0.129 0.991	0.008 0.006 0.008 0.007	0.023 0.018 0.024 0.262	-0.015 -0.012 -0.016 -0.252	1.49 1.50 1.50 1.03
CC1@HU	O111$\:-$H114 O113$\:-$H81 N1$\:-$H114 O113$\:-$H75 O111$\:-$C13	0.224 0.102 0.217 0.116 0.068	0.843 0.372 0.665 0.435 0.252	0.001 0.011 0.002 0.011 0.010	0.211 0.082 0.164 0.097 0.052	-0.210 -0.071 -0.161 -0.085 -0.042	1.00 1.15 1.01 1.14 1.23
CC1@CM	Cl109$\:-$H86 Cl109$\:-$H78 Cl109$\:-$H83 Cl116$\:-$H65 Cl116$\:-$H87 Cl116$\:-$H81 N1$\:-$H124 N2$\:-$H120	0.021 0.031 0.062 0.064 0.053 0.018 0.028 0.020	0.173 0.124 0.259 0.230 0.178 0.057 0.100 0.074	0.088 0.009 0.010 0.011 0.010 0.003 0.006 0.005	0.345 0.023 0.054 0.046 0.033 0.010 0.019 0.013	-0.257 -0.014 -0.044 -0.035 -0.023 -0.007 -0.013 -0.008	1.34 1.64 1.22 1.25 1.43 1.42 1.46 1.62
CC1@5-FU	O114$\:-$C19 N16$\:-$C44 N16$\:-$C13 O119$\:-$N2 N16$\:-$C38 O119$\:-$C37 N1$\:-$H117 F118-C36	0.047 0.063 0.077 0.038 0.064 0.119 0.106 0.118	0.170 0.217 0.240 0.246 0.230 0.406 0.357 0.516	0.009 0.009 0.008 0.006 0.011 0.011 0.010 0.018	0.034 0.045 0.052 0.030 0.046 0.091 0.079 0.110	-0.025 -0.036 -0.044 -0.024 -0.035 -0.080 -0.069 -0.092	1.36 1.25 1.18 1.25 1.31 1.13 1.14 1.19

### NCI analysis

**Fig. 8:**
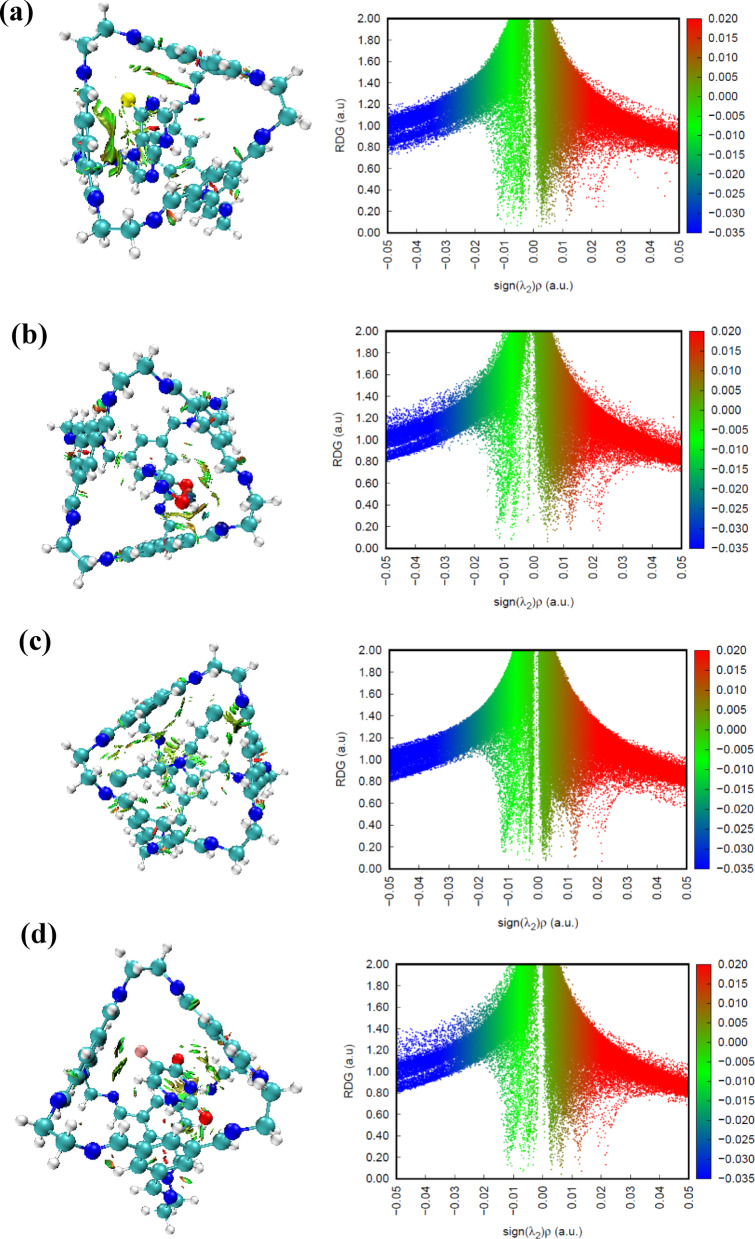

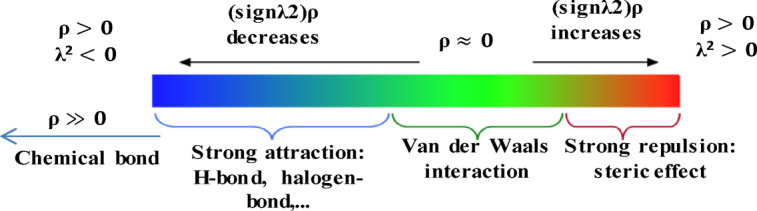
2D reduced density gradient (RDG) graph (right side) and 3D isosurface map (left side) for CC1@drug complexes: (**a**) CC1@6-MP, (**b**) CC1@HU, (**c**) CC1@CM, and (**d**) CC1@5-FU.

Non-covalent interaction (NCI) analysis was performed to explore the closed-shell interactions between the CC1 nanocage and the drug molecules using the reduced density gradient (RDG) method. Weak intermolecular interactions were visualized and characterized using both 2D RDG scatter plots and their corresponding 3D isosurfaces (Fig. 8). In the analysis, different interaction types are distinguished by the sign of the second eigenvalue (λ2) of the electron density Hessian matrix multiplied by the electron density (sign(λ2)ρ). Specifically, blue regions (sign(λ2)ρ < 0) correspond to strong attractive interactions such as hydrogen bonding, green regions (sign(λ2)ρ ≈ 0) indicate weak van der Waals forces, and red regions (sign(λ2)ρ > 0) signify strong steric repulsion^76^. For the CC1@drug complexes, prominent green regions are observed between the nanocage and the drug molecules in both the 3D isosurfaces and the 2D scatter plots (evident as green spikes at low density). This clearly indicates that weak van der Waals forces dominate the host–guest interactions. Such non-covalent interactions are essential for stabilizing the complexes without compromising the structural integrity of either component. Additionally, red spikes in the RDG scatter plots (Fig. 8), particularly in the range sign(λ2)ρ = 0.01 to 0.05 a.u., correspond to red patches on the 3D isosurfaces. These indicate steric repulsion, primarily within the constrained aromatic rings of the nanocage. This type of repulsion is common in such systems and does not destabilize the complex. Furthermore, these steric contours illustrate the specific spatial fit of the drug within the nanocage cavity, a factor that could potentially influence the drug release kinetics.

### Drug desorption

The calculated recovery times for the CC1@drug complexes at various temperatures are summarized in Table 7. The results clearly show that recovery time is closely linked to adsorption strength, highlighting a critical balance: shorter recovery times correspond to weaker adsorption and faster drug release, whereas longer recovery times indicate stronger interactions and more sustained retention within the nanocage. As expected, recovery time decreases with increasing temperature, promoting more rapid desorption. Notably, the CC1@CM complex exhibits the shortest recovery times (6.95 × 10⁻¹¹ s at 298 K, 3.77 × 10⁻¹¹ s at 348 K, and 2.38 × 10⁻¹¹ s at 398 K), reflecting the weakest adsorption and fastest release kinetics. In contrast, the CC1@5-FU complex shows the longest recovery times, consistent with its strongest adsorption, yet these times are still within a favorable range compared with previously reported nanocarrier systems^77–79^, ensuring effective and practical release. These findings demonstrate that the CC1 nanocage provides a tunable drug release system, where the balance between adsorption strength and recovery time can be optimized to achieve controlled and efficient drug delivery under different conditions. Furthermore, desorption times can be tuned or enhanced by strategies such as functionalizing the nanocage to strengthen interactions, optimizing the cavity size for better confinement, or applying external stimuli (temperature, pH, or light) to modulate release kinetics, enabling precise control over drug delivery^80–82^.

**Table 7:** Recovery times (τ) of CC1@drug complexes at different temperatures.

Compound	T(K)	τ (s)
CC1@6-MP	298 348 398	1.38 × 10^− 8^ 3.50 × 10^− 9^ 1.25 × 10^− 9^
CC1@HU	298 348 398	2.97 × 10^− 7^ 4.79 × 10^− 8^ 1.25 × 10^− 8^
CC1@CM	298 348 398	6.95 × 10^− 11^ 3.77 × 10^− 11^ 2.38 × 10^− 11^
CC1@5-FU	298 348 398	2.40 × 10^− 6^ 2.93 × 10^− 7^ 5.95 × 10^− 8^

## Conclusion

This study investigated the potential of the pristine CC1 nanocage as a drug delivery carrier for the anticancer drugs 6-MP, HU, CM, and 5-FU using density functional theory (DFT). The calculated adsorption energies confirmed that drug adsorption is exothermic and spontaneous. The magnitude of these energies and subsequent analyses revealed the interactions are physical (non-covalent) in nature. Frontier molecular orbital analysis showed a significant reduction in the HOMO-LUMO energy gap upon adsorption, with the CC1@5-FU complex exhibiting the largest decrease (48.70%), indicating a substantial increase in chemical reactivity. Charge transfer analysis consistently showed electron donation from the nanocage to the drug. The non-covalent, electrostatic character of the interactions was unequivocally confirmed by QTAIM and NCI analyses. Furthermore, the remarkably short recovery times, particularly for the CC1@CM complex (6.95 × 10^− 11^ s at 298 K), suggest efficient drug release is feasible. Collectively, these findings provide valuable atomic-level insights into the interaction mechanisms between the CC1 nanocage and anticancer drugs, underscoring its promise as a tunable platform for targeted drug delivery.

## Electronic Supplementary Material

Below is the link to the electronic supplementary material.

Supplementary Material 1

## Data Availability

Data sets generated during the current study are available from the corresponding author (Umme Hani) upon reasonable request.
