# Supplementary material for: Computational exploration of anticancer drug adsorption on a porous organic nanocage: Insights from density functional theory for targeted nanocarrier design
Source: Sci Rep. 2026 May 5;16:20646. doi: 10.1038/s41598-026-51498-8 (PMC13333833; doi:10.1038/s41598-026-51498-8)
Supplement: Supplementary file 1 — Supplementary Material 1 [file 41598_2026_51498_MOESM1_ESM.docx]

**Supporting information**

**Computational Exploration of Anticancer Drug Adsorption on a porous organic nanocage: Insights from Density Functional Theory for Targeted Nanocarrier Design**

Afrasim Moin^1^, Zahra Ali Ahmed Asiri^2^, Mona Al Hamod^3^, Noura Al Hamood^2^, Shahad Mari Salem Alshahrani^2^, Farhat Fatima^4^, Ali H. Alamri^2^, Adel alfatease^2^, Riyaz Ali M Osmani^2^, Mohamed Rahamathulla^2^, Umme Hani^2^*

[Ume.Haniii@proton.me](mailto:Ume.Haniii@proton.me)

^1^Department of Pharmaceutics, College of Pharmacy, University of Hail, Hail 81442, Saudi Arabia.

^2^Department of Pharmaceutics, College of Pharmacy, King Khalid University, Abha 62529 Saudi Arabia.

^3^Department of Pharmaceutics, Faculty of Pharmacy, Northern Border University, Rafhaa. 73213, Saudi Arabia.

^4^Department of Pharmaceutics, College of Pharmacy, Prince Sattam bin Abdulaziz University, Al-Kharj 11942, Saudi Arabia


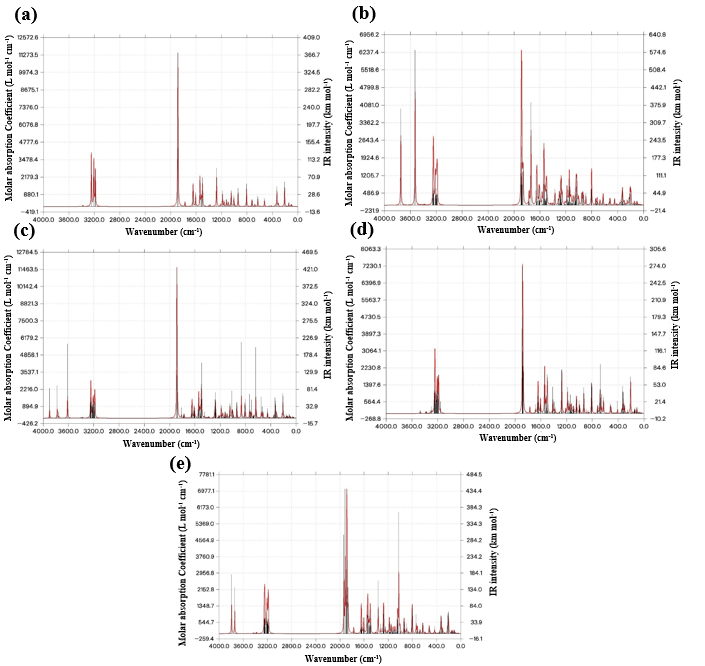


**Fig. S1.** IR spectra for (a) CC1 cage, (b) CC1@6-MP, (c) CC1@HU, (d) CC1@CM, and (e) CC1@5-FU.

**CC1 nanocage: Cartesian (Angstrom)**

**X Y Z**

N 0.98279833 -2.92059012 3.98098523

N 3.38920533 -3.11593912 2.29477023

N -0.74761867 5.02494088 4.28071923

N -4.62045167 -0.79448312 -1.92415977

N -4.49700567 -1.69632412 0.87585123

N 5.99814733 2.46684888 0.50712723

N 4.64266733 5.06493688 0.79898023

N -1.88007267 4.60600988 -4.04359877

N -2.16257667 2.56556588 5.06733923

N 3.52530133 -1.47708212 -3.90748577

N -0.97256667 6.48188688 -1.96332677

N 0.90182833 -1.06303612 -5.17797877

C -0.91187467 -1.68234012 3.17520223

C 3.98080733 -1.26579112 0.88077723

C -0.01367667 5.27236788 2.00630923

C -2.60964167 -0.03668812 -2.99823377

C -2.03733367 -1.70846712 2.36671423

C 3.53832533 -1.87717912 -0.29723777

C 1.33178533 5.01738788 2.29243823

C -2.94299867 1.31965688 -2.91898677

C -2.94364967 -0.64258312 2.37517523

C 3.71751033 -1.25599312 -1.52319677

C 2.29339433 5.11207288 1.29864323

C -2.12627667 2.28030188 -3.49451177

C -2.70490167 0.44887188 3.19545623

C 4.33819033 -0.00336112 -1.57573577

C 1.90834733 5.46043188 -0.00043477

C -0.95618967 1.88477588 -4.15153477

C -1.57207567 0.48732488 4.01585523

C 4.78024933 0.61288088 -0.41565777

C 0.57862833 5.71686988 -0.29593577

C -0.61417567 0.54416088 -4.23503477

C -0.68172467 -0.57478612 3.99871523

C 4.59544233 -0.02506712 0.81560523

C -0.38269767 5.61643088 0.71546223

C -1.44724767 -0.41605812 -3.65107377

C 0.01379233 -2.82994612 3.17437923

C 3.82292533 -1.93361812 2.18577723

C -1.03879167 5.20408188 3.06377323

C -3.48876767 -1.06755512 -2.41670277

C 1.82925233 -4.08989312 3.89765323

C 3.27378233 -3.67775112 3.62212023

C -1.83025967 4.97601988 5.23803823

C -5.40463767 -1.88086012 -1.38078577

C -4.15990067 -0.67670812 1.54262723

C 3.27266133 -1.93210812 -2.75549577

C 3.70950633 4.86811488 1.62854723

C -2.51356667 3.70136688 -3.42873277

C -5.71090267 -1.62548812 0.09355523

C 3.04584833 -2.21929112 -5.05214477

C 5.99834833 4.79791888 1.22492223

C -2.34465167 5.96901388 -3.91394177

C -1.32551267 1.63104588 4.91301223

C 5.46031633 1.91857388 -0.49682177

C 0.19881833 6.11244788 -1.66450477

C 0.60778533 0.14646188 -4.95783177

C -1.82290667 3.63861088 5.97518723

C 6.63963233 3.74888488 0.31891423

C -1.23995867 6.85091988 -3.33561477

C 2.12277733 -1.34569112 -5.89903077

H 1.50028833 -4.79532812 3.11984323

H 1.79683033 -4.60146312 4.86521323

H -2.24352267 -2.55659912 1.72219923

H 3.06341033 -2.85019412 -0.22722177

H 1.60242233 4.75410588 3.30963623

H -3.85864467 1.59740288 -2.40741177

H 3.60618033 -2.98118512 4.40646623

H 3.90104433 -4.57413212 3.66652723

H -3.39276767 1.28744588 3.22526523

H 4.47305933 0.46579888 -2.54474177

H 2.67543133 5.53345488 -0.76430577

H -0.33210367 2.65049088 -4.60036277

H -2.81511167 5.12534988 4.77042623

H -1.67058267 5.77341688 5.97119223

H 0.20281233 -0.57373212 4.62707823

H 4.95257533 0.47029688 1.71244123

H -1.41802767 5.82436188 0.46627623

H -1.16581367 -1.46090012 -3.73076977

H -4.90397967 -2.85553012 -1.48201377

H -0.20022367 -3.61398312 2.43271423

H 4.11954633 -1.34011412 3.06349823

H -2.07857767 5.33300888 2.72782423

H -3.10293267 -2.09739212 -2.45129077

H -6.35322267 -1.92057412 -1.92622677

H -4.76774367 0.24041888 1.54961623

H 2.71844333 -2.87141212 -2.61000477

H 3.90634733 4.51630888 2.65233323

H -3.40140867 3.92548288 -2.81874477

H -6.22233867 -0.65630012 0.19348723

H -6.38732867 -2.41174512 0.44445923

H 2.51144033 -3.13792512 -4.76684077

H 3.91114633 -2.49739812 -5.66278077

H 6.05390133 4.45549588 2.26921323

H 6.57120733 5.72667788 1.13423123

H -3.24122067 6.05171688 -3.28133377

H -2.59133167 6.33955288 -4.91432177

H -0.36804167 1.61315888 5.45486123

H 5.48128233 2.38356888 -1.49376777

H 1.00620733 6.07932088 -2.41127077

H 1.24436533 0.97265788 -5.30819477

H -0.84172967 3.49427988 6.45203323

H -2.58677567 3.67402888 6.75884623

H 6.59368233 4.09568788 -0.72440777

H 7.69192933 3.64645288 0.60370523

H -0.34742967 6.77700388 -3.97500577

H -1.58843667 7.88889888 -3.34599677

H 2.66050233 -0.43220412 -6.19446877

H 1.86034633 -1.90143312 -6.80521977

Item Value Threshold Converged

Maximum Force 0.000020 0.000450 YES

RMS Force 0.000006 0.000300 YES

Maximum Displacement 0.000914 0.001800 YES

RMS Displacement 0.000197 0.001200 YES

**CC1@6-MP nanocage: Cartesian (Angstrom)**

**X Y Z**

N 2.31332400 4.48082200 -2.32245600

N 2.63237300 2.05108200 -4.19333500

N -5.12344800 3.57945300 0.79321900

N 2.42104200 -0.06225300 4.85051400

N 2.46321500 2.82321900 3.87651100

N -2.53501400 -1.54087800 -5.20293000

N -4.90128700 -1.15057500 -3.51383300

N -2.62716600 -3.88151500 3.93920900

N -2.86346800 5.37409400 1.39187000

N 2.56831100 -4.19373800 -2.44192900

N -4.96260400 -2.52414800 2.71680000

N 2.69787100 -4.82626500 0.52219400

C 1.60172200 4.31909000 -0.02813700

C 1.24806400 0.06996000 -4.21672300

C -4.88083800 1.19515100 0.67223900

C 1.79398600 -2.03785300 3.64181100

C 1.94892200 3.86920300 1.23198300

C 2.20456100 -0.79497600 -3.68366100

C -4.86244400 1.16267800 -0.72054400

C 0.48754100 -2.03590000 4.11973400

C 1.01767800 3.88390600 2.26510400

C 1.88970500 -2.11984100 -3.44252700

C -4.82167800 -0.04275900 -1.39006500

C -0.41057400 -2.99390400 3.69857800

C -0.25372000 4.37066900 2.02859900

C 0.61346400 -2.59077100 -3.74270100

C -4.80018500 -1.23189200 -0.66496400

C -0.01700800 -3.94002500 2.75666300

C -0.60864300 4.83001300 0.76546700

C -0.32661600 -1.75065600 -4.29841300

C -4.81828800 -1.20991200 0.71353000

C 1.27174900 -3.94415300 2.26293100

C 0.32071700 4.80012700 -0.25732000

C -0.00271700 -0.41730100 -4.53252100

C -4.85681500 0.01231400 1.38103800

C 2.18789100 -3.01219300 2.74663000

C 2.57586000 4.17737600 -1.12933500

C 1.53408000 1.50270300 -4.44970200

C -4.96377500 2.48594100 1.38681500

C 2.73800300 -0.97381900 4.04848800

C 3.36203000 4.27193500 -3.33451900

C 2.82094700 3.47616100 -4.52568000

C -5.23892800 4.82702000 1.55997400

C 3.39505100 0.97530200 5.22379800

C 1.35509200 3.34507800 3.59764500

C 2.87091800 -3.03204700 -2.81216400

C -4.83064300 -0.07775300 -2.86676300

C -1.78509700 -3.00018200 4.23631800

C 2.73259900 2.35425000 5.24255000

C 3.55925300 -5.06701600 -1.79634800

C -4.93362400 -1.13520900 -4.98090600

C -3.97175500 -3.84508600 4.52596300

C -1.97886100 5.30460700 0.50301100

C -1.67472300 -2.25325200 -4.63115600

C -4.83522800 -2.47823700 1.46947400

C 1.62008100 -4.87476800 1.16809500

C -4.21145900 5.84265900 1.05518900

C -3.86058800 -2.08237700 -5.52149100

C -5.01572100 -3.81848600 3.40768700

C 2.94639100 -5.77434700 -0.57967700

H 4.22854100 3.76074000 -2.92654100

H 3.66966200 5.24583100 -3.69887400

H 2.92492200 3.46345400 1.40553600

H 3.17642800 -0.40149100 -3.45677200

H -4.89302300 2.09392500 -1.25030500

H 0.20309400 -1.27291400 4.81640600

H 1.89723800 3.93444100 -4.86641500

H 3.55077900 3.51658300 -5.32602700

H -0.99472600 4.38871600 2.80305800

H 0.38658500 -3.61359300 -3.52012300

H -4.78123100 -2.15649000 -1.20641700

H -0.74500400 -4.64681300 2.41278700

H -5.08766700 4.66809500 2.62347600

H -6.23463200 5.22102400 1.39627100

H 0.06847300 5.12210600 -1.24842800

H -0.75296600 0.21983800 -4.95594600

H -4.88053200 0.00599000 2.45243400

H 3.19974700 -3.04747900 2.40084400

H 4.24854900 0.99586800 4.55357600

H 3.51575300 3.74835500 -0.83144400

H 0.70203000 2.05245900 -4.87366900

H -4.89405800 2.41462800 2.46554400

H 3.71356700 -1.02689600 3.57964900

H 3.73864200 0.74829400 6.22682500

H 0.55982400 3.43056900 4.33071500

H 3.86024600 -2.61338100 -2.67123500

H -4.78312000 0.89139200 -3.34880200

H -2.00987100 -2.18973600 4.91936100

H 1.83270300 2.30972100 5.84953200

H 3.42509700 3.05516900 5.69486500

H 4.45411600 -4.52850000 -1.49659600

H 3.84058500 -5.82784200 -2.51565200

H -4.77063400 -0.14085100 -5.38561400

H -5.90737500 -1.49199400 -5.29393800

H -4.11744800 -2.98195000 5.16872000

H -4.10257500 -4.74918800 5.10757700

H -2.17236800 5.58436100 -0.52649500

H -1.86418900 -3.28147600 -4.34712800

H -4.74701900 -3.37089000 0.86173500

H 0.84417200 -5.59112700 0.92949000

H -4.34425900 5.98774500 -0.01322600

H -4.37695600 6.78508400 1.56360600

H -4.01223300 -3.07326900 -5.10388900

H -3.94840900 -2.13289400 -6.60000900

H -4.84028500 -4.65222200 2.73437300

H -6.00131700 -3.91618100 3.84624500

H 2.03706200 -6.28064700 -0.88626200

H 3.65832700 -6.51108500 -0.22541400

C -2.15928037 -0.52295393 -0.50695005

C -0.00984863 0.51220320 -1.07869960

C -0.52226973 1.71525811 -0.93092834

C -1.98801308 1.89006566 -0.59709237

H -2.66169439 -1.38953378 -0.13075925

H -0.35074102 -1.46579667 -0.75558302

N 0.51377156 2.72515683 -1.11932593

N 1.45559489 0.61326767 -1.11084694

C 1.72700517 1.88778857 -0.97005233

H 0.46352778 3.13322307 -2.03089463

H 2.70301484 2.27501725 -0.76423915

N -2.73730697 0.65082322 -0.36059339

S -2.65809994 3.30230465 -0.49622825

N -0.83606404 -0.70237719 -1.18178986

Item Value Threshold Converged

Maximum Force 0.000013 0.000450 YES

RMS Force 0.000002 0.000300 YES

Maximum Displacement 0.000459 0.001800 YES

RMS Displacement 0.000108 0.001200 YES

**CC1@HU nanocage: Cartesian (Angstrom)**

**X Y Z**

N 0.98279833 -2.92059012 3.98098523

N 3.38920533 -3.11593912 2.29477023

N -0.74761867 5.02494088 4.28071923

N -4.62045167 -0.79448312 -1.92415977

N -4.49700567 -1.69632412 0.87585123

N 5.99814733 2.46684888 0.50712723

N 4.64266733 5.06493688 0.79898023

N -1.88007267 4.60600988 -4.04359877

N -2.16257667 2.56556588 5.06733923

N 3.52530133 -1.47708212 -3.90748577

N -0.97256667 6.48188688 -1.96332677

N 0.90182833 -1.06303612 -5.17797877

C -0.91187467 -1.68234012 3.17520223

C 3.98080733 -1.26579112 0.88077723

C -0.01367667 5.27236788 2.00630923

C -2.60964167 -0.03668812 -2.99823377

C -2.03733367 -1.70846712 2.36671423

C 3.53832533 -1.87717912 -0.29723777

C 1.33178533 5.01738788 2.29243823

C -2.94299867 1.31965688 -2.91898677

C -2.94364967 -0.64258312 2.37517523

C 3.71751033 -1.25599312 -1.52319677

C 2.29339433 5.11207288 1.29864323

C -2.12627667 2.28030188 -3.49451177

C -2.70490167 0.44887188 3.19545623

C 4.33819033 -0.00336112 -1.57573577

C 1.90834733 5.46043188 -0.00043477

C -0.95618967 1.88477588 -4.15153477

C -1.57207567 0.48732488 4.01585523

C 4.78024933 0.61288088 -0.41565777

C 0.57862833 5.71686988 -0.29593577

C -0.61417567 0.54416088 -4.23503477

C -0.68172467 -0.57478612 3.99871523

C 4.59544233 -0.02506712 0.81560523

C -0.38269767 5.61643088 0.71546223

C -1.44724767 -0.41605812 -3.65107377

C 0.01379233 -2.82994612 3.17437923

C 3.82292533 -1.93361812 2.18577723

C -1.03879167 5.20408188 3.06377323

C -3.48876767 -1.06755512 -2.41670277

C 1.82925233 -4.08989312 3.89765323

C 3.27378233 -3.67775112 3.62212023

C -1.83025967 4.97601988 5.23803823

C -5.40463767 -1.88086012 -1.38078577

C -4.15990067 -0.67670812 1.54262723

C 3.27266133 -1.93210812 -2.75549577

C 3.70950633 4.86811488 1.62854723

C -2.51356667 3.70136688 -3.42873277

C -5.71090267 -1.62548812 0.09355523

C 3.04584833 -2.21929112 -5.05214477

C 5.99834833 4.79791888 1.22492223

C -2.34465167 5.96901388 -3.91394177

C -1.32551267 1.63104588 4.91301223

C 5.46031633 1.91857388 -0.49682177

C 0.19881833 6.11244788 -1.66450477

C 0.60778533 0.14646188 -4.95783177

C -1.82290667 3.63861088 5.97518723

C 6.63963233 3.74888488 0.31891423

C -1.23995867 6.85091988 -3.33561477

C 2.12277733 -1.34569112 -5.89903077

H 1.50028833 -4.79532812 3.11984323

H 1.79683033 -4.60146312 4.86521323

H -2.24352267 -2.55659912 1.72219923

H 3.06341033 -2.85019412 -0.22722177

H 1.60242233 4.75410588 3.30963623

H -3.85864467 1.59740288 -2.40741177

H 3.60618033 -2.98118512 4.40646623

H 3.90104433 -4.57413212 3.66652723

H -3.39276767 1.28744588 3.22526523

H 4.47305933 0.46579888 -2.54474177

H 2.67543133 5.53345488 -0.76430577

H -0.33210367 2.65049088 -4.60036277

H -2.81511167 5.12534988 4.77042623

H -1.67058267 5.77341688 5.97119223

H 0.20281233 -0.57373212 4.62707823

H 4.95257533 0.47029688 1.71244123

H -1.41802767 5.82436188 0.46627623

H -1.16581367 -1.46090012 -3.73076977

H -4.90397967 -2.85553012 -1.48201377

H -0.20022367 -3.61398312 2.43271423

H 4.11954633 -1.34011412 3.06349823

H -2.07857767 5.33300888 2.72782423

H -3.10293267 -2.09739212 -2.45129077

H -6.35322267 -1.92057412 -1.92622677

H -4.76774367 0.24041888 1.54961623

H 2.71844333 -2.87141212 -2.61000477

H 3.90634733 4.51630888 2.65233323

H -3.40140867 3.92548288 -2.81874477

H -6.22233867 -0.65630012 0.19348723

H -6.38732867 -2.41174512 0.44445923

H 2.51144033 -3.13792512 -4.76684077

H 3.91114633 -2.49739812 -5.66278077

H 6.05390133 4.45549588 2.26921323

H 6.57120733 5.72667788 1.13423123

H -3.24122067 6.05171688 -3.28133377

H -2.59133167 6.33955288 -4.91432177

H -0.36804167 1.61315888 5.45486123

H 5.48128233 2.38356888 -1.49376777

H 1.00620733 6.07932088 -2.41127077

H 1.24436533 0.97265788 -5.30819477

H -0.84172967 3.49427988 6.45203323

H -2.58677567 3.67402888 6.75884623

H 6.59368233 4.09568788 -0.72440777

H 7.69192933 3.64645288 0.60370523

H -0.34742967 6.77700388 -3.97500577

H -1.58843667 7.88889888 -3.34599677

H 2.66050233 -0.43220412 -6.19446877

H 1.86034633 -1.90143312 -6.80521977

N -0.71577488 0.61715741 0.16897738

C 0.56877412 0.57346912 0.88236732

O 0.84917784 -0.41149178 1.61366503

N 1.52576950 1.68036294 0.74149180

O 2.25557051 1.79674210 1.88317757

H 2.88054708 2.51961153 1.79117723

H 1.02843896 2.53190110 0.57552468

H -0.60784251 1.13343977 -0.68061259

H -1.40177951 1.06218246 0.74460778

Item Value Threshold Converged

Maximum Force 0.000044 0.000450 YES

RMS Force 0.000004 0.000300 YES

Maximum Displacement 0.002821 0.001800 NO

RMS Displacement 0.000477 0.001200 YES

Predicted change in Energy=-6.730724D-08

**CC1@CM nanocage: Cartesian (Angstrom)**

**X Y Z**

N 2.28412009 -2.52022464 4.38712970

N 4.69052709 -2.71557364 2.70091470

N 0.55370309 5.42530636 4.68686370

N -3.31912991 -0.39411764 -1.51801530

N -3.19568391 -1.29595864 1.28199570

N 7.29946909 2.86721436 0.91327170

N 5.94398909 5.46530236 1.20512470

N -0.57875091 5.00637536 -3.63745430

N -0.86125491 2.96593136 5.47348370

N 4.82662309 -1.07671664 -3.50134130

N 0.32875509 6.88225236 -1.55718230

N 2.20315009 -0.66267064 -4.77183430

C 0.38944709 -1.28197464 3.58134670

C 5.28212909 -0.86542564 1.28692170

C 1.28764509 5.67273336 2.41245370

C -1.30831991 0.36367736 -2.59208930

C -0.73601191 -1.30810164 2.77285870

C 4.83964709 -1.47681364 0.10890670

C 2.63310709 5.41775336 2.69858270

C -1.64167691 1.72002236 -2.51284230

C -1.64232791 -0.24221764 2.78131970

C 5.01883209 -0.85562764 -1.11705230

C 3.59471609 5.51243836 1.70478770

C -0.82495491 2.68066736 -3.08836730

C -1.40357991 0.84923736 3.60160070

C 5.63951209 0.39700436 -1.16959130

C 3.20966909 5.86079736 0.40570970

C 0.34513209 2.28514136 -3.74539030

C -0.27075391 0.88769036 4.42199970

C 6.08157109 1.01324636 -0.00951330

C 1.87995009 6.11723536 0.11020870

C 0.68714609 0.94452636 -3.82889030

C 0.61959709 -0.17442064 4.40485970

C 5.89676409 0.37529836 1.22174970

C 0.91862409 6.01679636 1.12160670

C -0.14592591 -0.01569264 -3.24492930

C 1.31511409 -2.42958064 3.58052370

C 5.12424709 -1.53325264 2.59192170

C 0.26253009 5.60444736 3.46991770

C -2.18744591 -0.66718964 -2.01055830

C 3.13057409 -3.68952764 4.30379770

C 4.57510409 -3.27738564 4.02826470

C -0.52893791 5.37638536 5.64418270

C -4.10331591 -1.48049464 -0.97464130

C -2.85857891 -0.27634264 1.94877170

C 4.57398309 -1.53174264 -2.34935130

C 5.01082809 5.26848036 2.03469170

C -1.21224491 4.10173236 -3.02258830

C -4.40958091 -1.22512264 0.49969970

C 4.34717009 -1.81892564 -4.64600030

C 7.29967009 5.19828436 1.63106670

C -1.04332991 6.36937936 -3.50779730

C -0.02419091 2.03141136 5.31915670

C 6.76163809 2.31893936 -0.09067730

C 1.50014009 6.51281336 -1.25836030

C 1.90910709 0.54682736 -4.55168730

C -0.52158491 4.03897636 6.38133170

C 7.94095409 4.14925036 0.72505870

C 0.06136309 7.25128536 -2.92947030

C 3.42409909 -0.94532564 -5.49288630

H 2.80161009 -4.39496264 3.52598770

H 3.09815209 -4.20109764 5.27135770

H -0.94220091 -2.15623364 2.12834370

H 4.36473209 -2.44982864 0.17892270

H 2.90374409 5.15447136 3.71578070

H -2.55732291 1.99776836 -2.00126730

H 4.90750209 -2.58081964 4.81261070

H 5.20236609 -4.17376664 4.07267170

H -2.09144591 1.68781136 3.63140970

H 5.77438109 0.86616436 -2.13859730

H 3.97675309 5.93382036 -0.35816130

H 0.96921809 3.05085636 -4.19421830

H -1.51378991 5.52571536 5.17657070

H -0.36926091 6.17378236 6.37733670

H 1.50413409 -0.17336664 5.03322270

H 6.25389709 0.87066236 2.11858570

H -0.11670591 6.22472736 0.87242070

H 0.13550809 -1.06053464 -3.32462530

H -3.60265791 -2.45516464 -1.07586930

H 1.10109809 -3.21361764 2.83885870

H 5.42086809 -0.93974864 3.46964270

H -0.77725591 5.73337436 3.13396870

H -1.80161091 -1.69702664 -2.04514630

H -5.05190091 -1.52020864 -1.52008230

H -3.46642191 0.64078436 1.95576070

H 4.01976509 -2.47104664 -2.20386030

H 5.20766909 4.91667436 3.05847770

H -2.10008691 4.32584836 -2.41260030

H -4.92101691 -0.25593464 0.59963170

H -5.08600691 -2.01137964 0.85060370

H 3.81276209 -2.73755964 -4.36069630

H 5.21246809 -2.09703264 -5.25663630

H 7.35522309 4.85586136 2.67535770

H 7.87252909 6.12704336 1.54037570

H -1.93989891 6.45208236 -2.87518930

H -1.29000991 6.73991836 -4.50817730

H 0.93328009 2.01352436 5.86100570

H 6.78260409 2.78393436 -1.08762330

H 2.30752909 6.47968636 -2.00512630

H 2.54568709 1.37302336 -4.90205030

H 0.45959209 3.89464536 6.85817770

H -1.28545391 4.07439436 7.16499070

H 7.89500409 4.49605336 -0.31826330

H 8.99325109 4.04681836 1.00984970

H 0.95389209 7.17736936 -3.56886130

H -0.28711491 8.28926436 -2.93985230

H 3.96182409 -0.03183864 -5.78832430

H 3.16166809 -1.50106764 -6.39907530

Cl -1.70514730 0.31605112 0.46374043

C -0.49024454 1.36081725 -0.26432209

C 0.83340899 1.22687640 0.51130745

N 1.84812891 2.09949356 -0.09679023

C 1.38893154 3.49497231 -0.04505185

C 3.11161637 1.97164094 0.64358342

C 4.17465628 2.88581130 0.00652872

Cl 5.68740317 2.73273605 0.89296248

H -0.82449024 2.37657388 -0.22666218

H -0.33707453 1.07329372 -1.28352235

H 0.68023899 1.51439993 1.53050771

H 1.16765469 0.21111977 0.47364754

H 2.12753720 4.13014262 -0.48768077

H 0.46925019 3.58803510 -0.58396328

H 1.23576154 3.78249583 0.97414841

H 3.44586207 0.95588430 0.60592351

H 2.95844636 2.25916446 1.66278368

H 4.32782628 2.59828777 -1.01267154

H 3.84041058 3.90156793 0.04418863

Item Value Threshold Converged

Maximum Force 0.000024 0.000450 YES

RMS Force 0.000003 0.000300 YES

Maximum Displacement 0.001845 0.001800 NO

RMS Displacement 0.000267 0.001200 YES

**CC1@5-FU nanocage: Cartesian (Angstrom)**

**X Y Z**

N 0.60566266 -2.83183843 3.78140909

N 3.01206966 -3.02718743 2.09519409

N -1.12475434 5.11369257 4.08114309

N -4.99758734 -0.70573143 -2.12373591

N -4.87414134 -1.60757243 0.67627509

N 5.62101166 2.55560057 0.30755109

N 4.26553166 5.15368857 0.59940409

N -2.25720834 4.69476157 -4.24317491

N -2.53971234 2.65431757 4.86776309

N 3.14816566 -1.38833043 -4.10706191

N -1.34970234 6.57063857 -2.16290291

N 0.52469266 -0.97428443 -5.37755491

C -1.28901034 -1.59358843 2.97562609

C 3.60367166 -1.17703943 0.68120109

C -0.39081234 5.36111957 1.80673309

C -2.98677734 0.05206357 -3.19780991

C -2.41446934 -1.61971543 2.16713809

C 3.16118966 -1.78842743 -0.49681391

C 0.95464966 5.10613957 2.09286209

C -3.32013434 1.40840857 -3.11856291

C -3.32078534 -0.55383143 2.17559909

C 3.34037466 -1.16724143 -1.72277291

C 1.91625866 5.20082457 1.09906709

C -2.50341234 2.36905357 -3.69408791

C -3.08203734 0.53762357 2.99588009

C 3.96105466 0.08539057 -1.77531191

C 1.53121166 5.54918357 -0.20001091

C -1.33332534 1.97352757 -4.35111091

C -1.94921134 0.57607657 3.81627909

C 4.40311366 0.70163257 -0.61523391

C 0.20149266 5.80562157 -0.49551191

C -0.99131134 0.63291257 -4.43461091

C -1.05886034 -0.48603443 3.79913909

C 4.21830666 0.06368457 0.61602909

C -0.75983334 5.70518257 0.51588609

C -1.82438334 -0.32730643 -3.85064991

C -0.36334334 -2.74119443 2.97480309

C 3.44578966 -1.84486643 1.98620109

C -1.41592734 5.29283357 2.86419709

C -3.86590334 -0.97880343 -2.61627891

C 1.45211666 -4.00114143 3.69807709

C 2.89664666 -3.58899943 3.42254409

C -2.20739534 5.06477157 5.03846209

C -5.78177334 -1.79210843 -1.58036191

C -4.53703634 -0.58795643 1.34305109

C 2.89552566 -1.84335643 -2.95507191

C 3.33237066 4.95686657 1.42897109

C -2.89070234 3.79011857 -3.62830891

C -6.08803834 -1.53673643 -0.10602091

C 2.66871266 -2.13053943 -5.25172091

C 5.62121266 4.88667057 1.02534609

C -2.72178734 6.05776557 -4.11351791

C -1.70264834 1.71979757 4.71343609

C 5.08318066 2.00732557 -0.69639791

C -0.17831734 6.20119957 -1.86408091

C 0.23064966 0.23521357 -5.15740791

C -2.20004234 3.72736257 5.77561109

C 6.26249666 3.83763657 0.11933809

C -1.61709434 6.93967157 -3.53519091

C 1.74564166 -1.25693943 -6.09860691

H 1.12315266 -4.70657643 2.92026709

H 1.41969466 -4.51271143 4.66563709

H -2.62065834 -2.46784743 1.52262309

H 2.68627466 -2.76144243 -0.42679791

H 1.22528666 4.84285757 3.11006009

H -4.23578034 1.68615457 -2.60698791

H 3.22904466 -2.89243343 4.20689009

H 3.52390866 -4.48538043 3.46695109

H -3.76990334 1.37619757 3.02568909

H 4.09592366 0.55455057 -2.74431791

H 2.29829566 5.62220657 -0.96388191

H -0.70923934 2.73924257 -4.79993891

H -3.19224734 5.21410157 4.57085009

H -2.04771834 5.86216857 5.77161609

H -0.17432334 -0.48498043 4.42750209

H 4.57543966 0.55904857 1.51286509

H -1.79516334 5.91311357 0.26670009

H -1.54294934 -1.37214843 -3.93034591

H -5.28111534 -2.76677843 -1.68158991

H -0.57735934 -3.52523143 2.23313809

H 3.74241066 -1.25136243 2.86392209

H -2.45571334 5.42176057 2.52824809

H -3.48006834 -2.00864043 -2.65086691

H -6.73035834 -1.83182243 -2.12580291

H -5.14487934 0.32917057 1.35004009

H 2.34130766 -2.78266043 -2.80958091

H 3.52921166 4.60506057 2.45275709

H -3.77854434 4.01423457 -3.01832091

H -6.59947434 -0.56754843 -0.00608891

H -6.76446434 -2.32299343 0.24488309

H 2.13430466 -3.04917343 -4.96641691

H 3.53401066 -2.40864643 -5.86235691

H 5.67676566 4.54424757 2.06963709

H 6.19407166 5.81542957 0.93465509

H -3.61835634 6.14046857 -3.48090991

H -2.96846734 6.42830457 -5.11389791

H -0.74517734 1.70191057 5.25528509

H 5.10414666 2.47232057 -1.69334391

H 0.62907166 6.16807257 -2.61084691

H 0.86722966 1.06140957 -5.50777091

H -1.21886534 3.58303157 6.25245709

H -2.96391134 3.76278057 6.55927009

H 6.21654666 4.18443957 -0.92398391

H 7.31479366 3.73520457 0.40412909

H -0.72456534 6.86575557 -4.17458191

H -1.96557234 7.97765057 -3.54557291

H 2.28336666 -0.34345243 -6.39404491

H 1.48321066 -1.81268143 -7.00479591

C 0.92025715 -0.42872667 0.25582153

C 0.53165271 1.94180001 -0.08772354

C -1.34361619 0.38106812 -0.42801112

H -1.43881322 2.39653295 -0.37065383

C -0.56549333 -0.64765524 -0.02977386

O 0.80972448 3.08330838 0.36305943

N -0.79045657 1.71830237 -0.71653955

N 1.54333713 0.87048045 -0.00233177

H 2.18191153 1.06525917 0.74217074

F -1.10698335 -1.87308481 0.13639150

O 1.61076930 -1.37001547 0.72565015

H -2.39683550 0.22326417 -0.53158228

Item Value Threshold Converged

Maximum Force 0.000014 0.000450 YES

RMS Force 0.000002 0.000300 YES

Maximum Displacement 0.001616 0.001800 YES

RMS Displacement 0.000263 0.001200 YES
